# Editorial: The mechanism of tumor evolution and microenvironmental changes of genitourinary oncology in clinical diagnosis and treatment

**DOI:** 10.3389/fonc.2023.1272984

**Published:** 2023-08-29

**Authors:** Jiahe Lu, Wen-Hao Xu, Hailiang Zhang, Dingwei Ye

**Affiliations:** ^1^ Department of Urology, Fudan University Shanghai Cancer Center, Shanghai, China; ^2^ Department of Oncology, Shanghai Medical College, Fudan University, Shanghai, China; ^3^ Shanghai Genitourinary Cancer Institute, Shanghai, China

**Keywords:** the tumor microenvironment (TME), genitourinary (GU), renal cell carcinoma (RCC), prostate adenocarcinoma (PRAD), bladder cancer (BCa)

The tumor microenvironment (TME) encompasses the immediate surroundings in which tumor cells undergo formation and progression. This includes the peritumoral blood vessels, immune cells, fibroblasts, bone marrow-derived inflammatory cells, diverse signaling chemicals, and extracellular matrix (ECM) (1). The tumor microenvironment is closely related to tumorigenesis and immune escape. Consequently, there has been a paradigm shift in cancer research and treatment, transitioning from a focus solely on cancer cells to a more comprehensive approach centered upon the TME. This Research Topic aims to have a thorough examination of the numerous interactions occurring between genitourinary tumor cells and their adjacent microenvironment in order to comprehend the diverse underlying mechanisms affecting genitourinary tumor diagnosis and treatment.

## Renal cell carcinoma (RCC)

One of the defining characteristics of clear cell RCC (ccRCC) is the occurrence of deletion, mutation, and/or promoter methylation on chromosome 3p, resulting in the functional inactivation of the Von Hippel-Lindau (VHL) tumor suppressor gene. This inactivation subsequently leads to the abnormal accumulation of hypoxia-inducible factor (HIF) and the activation of the angiogenic pathway (2). Several additional genomic abnormalities have been identified in relation to disease progression and invasiveness. These include mutations in genes associated with the 3p region, namely PBRM1, SETD2, and BAP1. Additionally, deletions at the 9p21 locus can lead to the loss of CDKN2A and CDKN2B genes. Furthermore, alterations in KDM5C, TP53, MTOR, or PTEN have also been found to be associated with the extent of disease progression and invasiveness (3). Pharmacological interventions for RCC encompass many approaches such as cytokines, molecularly targeted therapeutics, and innovative immunosuppressive agents. Notably, these treatments mostly focus on modulating the tumor microenvironment rather than directly targeting the RCC tumor cells. ccRCC is a tumor form that exhibits significant inflammation, as evidenced by its high immune infiltration score in pan-cancer analysis (4).

The presence of unique yet fluctuating levels of vascular density, immune cell infiltration, and PD-L1 expression in ccRCC necessitates the use of inhibitors targeting the vascular endothelial growth factor (VEGF) pathway and the PD-L1 axis. Administering these inhibitors, either individually or in combination, has been shown to greatly enhance the clinical results of patients with advanced RCC. Nevertheless, there are instances where certain individuals exhibit a lack of response to this particular treatment, and it is worth noting that these treatments are accompanied by notable levels of toxicity. Hence, it is imperative to acquire a more comprehensive comprehension of the molecular underpinnings that contribute to the clinical variability observed in individuals with advanced RCC. Such understanding is crucial for devising effective treatment regimens and elucidating the mechanisms underlying resistance to therapies.

Due to its insensitivity to radiotherapy and chemotherapy, the primary treatment options for advanced ccRCC, which is the most prevalent and malignant subtype of RCC, are palliative tumor resection, targeted therapy, and immunotherapy (5). Fan et al. identified a novel immune subtyperelated prognostic signature of ccRCC associated with the expression of vacuole membrane protein 1. Guo et al. focused on a rare type of RCC, Xp11.2 translocation RCC, and investigated the clinical and pathological heterogeneity of its different fusion subtypes. Liu et al. showed that not only are sarcopenia and inflammation associated with tumor progression, leading to poor survival of RCC patients, but tumormediated inflammation may in turn exacerbate muscle wasting and further create a tumor-penetrating vicious cycle between sarcopenia and inflammation. Zhang et al. showed that patients with a high claudinTME related (CTR) risk signature may be more sensitive to immune checkpoint blockade.

It is noteworthy that the majority of the literature indicates a lack of correlation between RCC and immunodeficiency generated by the human immunodeficiency virus (HIV). Zhu et al. comprehensively reviewed the epidemiology, risk factors, and diagnostic approaches pertaining to RCC in individuals affected by HIV. Additionally, the authors offered significant perspectives on the management strategies for RCC patients with HIV.

## Prostate adenocarcinoma (PRAD)

PRAD is predominantly a hormone-mediated disease with androgen receptor (AR) signaling driving cell proliferation. The standard of care for PRAD is castration therapy or androgen deprivation therapy (ADT). However, ADT-treated patients will inevitably develop treatment resistance. Combining ADT with other therapeutic agents is therefore of considerable interest. Wang et al. conducted a systematic review of doublet and triplet therapies for hormone-sensitive metastatic prostate cancer and concluded that both showed a significant increase in overall survival, although triplet therapies may be less safe. Post-transcriptional modifications may affect the initiation and progression of tumors. The prognostic value of N6-methyladenosine (m6A) regulators in patients with metastatic prostate cancer was demonstrated by Liu et al. and Guo et al. examined the targeting mechanism of the ubiquitin-specific protease family as potential PRAD therapies.

The homeostasis and growth of the TME are governed by the complex intercellular communication, which includes extracellular metabolites. Using byproducts of sugar metabolism, cancer cells can co-opt tumorinfiltrating immune cells (6). Liu et al. developed a metabolic prediction model with a 12-mRNA signature that predicts the progression of PRAD patients. In addition, they discover that the metabolic enzyme myo-inositol oxygenase is associated with the DNA damage repair process in PRAD and plays a significant role in the aberrant immune infiltration of the TME.

## Bladder cancer (BCa)

BCa has the highest incidence rate among genitourinary malignancies. Chemotherapy is currently the mainstay treatment for advanced BCa. As with other malignancies, numerous immunotherapy clinical trials for BCa are currently ongoing. However, the TME of BCa and the immune signature within it remain unclear, making it difficult to predict the efficacy of immunotherapy. Yang et al. discovered that adenylate cyclase 2 methylation is a reliable biomarker for the diagnosis and immunotherapy of patients with BCa. Integrins, a class of cell surface adhesion molecules with signal transduction functions, serve a crucial role in virtually every phase of tumor proliferation and metastasis. Recent research indicates that homoharringtonine may inhibit the growth of bladder cancer by inactivating the integrin 5/1-FAK/Src axis (7). Collagen induces senescence in tumor cells by activating the p53/p21 pathway via the integrin 1/AKT axis (8). Tu et al. identified three integrin subunit genes that may serve as bladder cancer prognostic markers. Overall, the complex effects of integrins and other adhesion factors in the tumor microenvironment, such as the equilibrium between the formation of a protective tumor shield and the recruitment of immune cells, require further investigation.

## Other genitourinary cancers

Systemic inflammation has prognostic value for patient overall survival, as mentioned previously. Wang et al. hypothesized that the same holds true for testicular germ cell tumor. Neutrophil-to-lymphocyte ratio (NLR) and systemic immune-inflammation index (SII) may be cost-effective and more accessible future markers.

In numerous forms of cancer, including penile cancer, cancer-associated fibroblasts (CAFs) are a vital component of the tumor microenvironment. CAFs contribute significantly to tumor progression, angiogenesis, invasion, and metastasis. Cury et al. demonstrated that a drug that targets matrix metalloproteinases can modulate CAFs, thereby expanding treatment options for penile cancer.

## Author contributions

JL: Writing – original draft. WX: Writing – original draft. HZ: Writing – review & editing. DY: Writing – review & editing.
